# Familial and lifestyle factors related to physical activity in elementary school students: a cross-sectional study based on a nationally representative survey in Japan

**DOI:** 10.1186/s12887-023-04162-3

**Published:** 2023-07-04

**Authors:** Noriko Motoki, Haruka Morota, Takumi Shibazaki, Chizuko Nakamura, Yozo Nakazawa

**Affiliations:** Department of Pediatrics, Shinshu University School of Medicine, Asahi 3-1-1, Matsumoto, Nagano, 390-8621 Japan

**Keywords:** Exercise, Moderate-to-vigorous physical activity, Organized sports, Children, Elementary school, Socioeconomic factor

## Abstract

**Purpose:**

The decrease in physical activity (PA) among children has become a global concern. Since the analysis of sociodemographic factors as determinants of exercise habits has been inconclusive, this study investigated the factors related to participation in organized sports and moderate-to-vigorous PA (MVPA) levels.

**Methods:**

Cross-sectional data from the Sports-Life Survey conducted in 2019 by the Sasagawa Sports Foundation were used. Data on the gender, age, grade, annual household income, family members, and lifestyle habits of elementary school children as well as information on participation in organized sports and MVPA were collected by written questionnaires. Multiple logistic regression models were applied to calculate the adjusted odds ratio and 95% confidence interval for the association of each variable with participation in organized sports and frequent MVPA (≥ 60 min/day for ≥ 5 days/week).

**Results:**

A total of 1,197 participants were included in the analysis. Whereas 1,053 (88.2%) students expressed a like for PA, only 725 students (60.8%) actually took part in organized sports. Organized sports participation was significantly associated with gender, grade, population density, household income, daily breakfast, lower screen time, and frequent exercise with parents (all *P* < 0.05). We observed that 12.3% of participants met the frequent MVPA level, which was significantly related to lower screen time and exercise habits with parents (both *P* < 0.05).

**Conclusions:**

Social and family factors may be strong determinants of engagement in PA among Japanese elementary school-aged children. Parental involvement appears particularly important for promoting PA among youths.

**Supplementary Information:**

The online version contains supplementary material available at 10.1186/s12887-023-04162-3.

## What is known

Social and family factors reportedly influence children's preference and participation in physical activities. However, no large-scale studies have been conducted on representative Japanese populations using such personal and difficult-to-answer information as family factors and household income.

## What is new

The present study targeted elementary school students throughout Japan and revealed that social and family factors may be strong determinants of engagement in physical activity. Parental involvement appears particularly important for promoting physical activity among youths.

## Introduction

Exercise and physical activity (PA) in children are important for healthy physical and mental development and subsequent adult health. The World Health Organization (WHO) 2020 guidelines on PA recommend an average of 60 min of moderate-to-vigorous PA (MVPA) daily for children and adolescents, which can provide many of the benefits of PA [1]. Acquiring exercise habits in childhood also leads to continued exercise routines in adults to provide positive effects on adiposity [2, 3], cardiovascular disease [4], bone health [3, 5], cognitive function [6, 7], and mental health [8].

The development of childhood motor skills is important for subsequent health, with older children having better motor coordination skills more likely to engage in increased PA than those with lesser coordination skills [9]. Motor coordination is significantly higher after 6–9 years of continuous organized sports participation compared with sporadic or no participation [10]. Children who find themselves superior in the skills required for a specific organized sport more frequently enjoy the activity and are more likely to stay in the sport than those who do not [11]. As such, elementary school students who participate in organized sports may benefit from motor skill acquisition and lifelong attention to the activity.

The decrease in PA among children has become a recent global issue [1]. In many countries and regions, the majority of adolescents do not meet current WHO PA guidelines. Therefore, increasing the activity of school-aged children requires the urgent expansion of known effective policies and programs [12]. A primary analysis of the of the national Sports-Life Survey conducted by the Sasagawa Sports Foundation, a large-scale survey in Japan, found that children aged 4 to 11 years showed a gradually decreasing frequency of sports and exercise engagement. Moreover, the proportion of young people between the ages of 12 and 21 years who have not exercised at all in the past year tended to increase [13]. The Japan Sports Agency has reported that the physical ability of elementary and junior high school students has been declining since 2019, citing reasons related to a decrease in exercise time, an increase in screen time on electronic devices, and a rise in childhood obesity [14, 15]. In contrast, more time spent participating in organized sports has been associated with higher amounts of MVPA in adolescents, the results of which support the use of sports participation as an effective strategy to increase PA levels [16].

Socioeconomic background factors, including insufficient financial resources and opposition from parents, have been reported as barriers for students who do not participate in sports [16]. Sawa et al. described that Japanese elementary school students who did not have close friends, engaged in long periods of screen time, and lacked parental communication had significant links with both a dislike and lack of PA [17]. Several other studies have supported the idea that social and family factors influence children's preference and participation in PA. However, those had a limited geographical target area (rural cities) and did not contain such personal and difficult-to-answer information as family factors and household income [17, 18]. Also considering reports of prefectural disparities in health and PA indicators in Japan [19, 20], the findings of surveys limited to small regions may not be representative of Japan as a whole.

The present study targeted elementary school students across 225 residential areas throughout Japan as a secondary analysis of the aforementioned national Sports-Life Survey [13] and aimed to investigate the relationships of participation in organized sports activities and frequent MVPA with social background, family factors, and child lifestyles towards improving health later in life.

## Methods

### Participants and data collection

This study was a secondary analysis of the national Sports-Life Survey conducted by the Sasagawa Sports Foundation in June-July 2019. Survey participants were selected by a stratified two-stage random sampling method with a set sample size of 2,400 participants. For geographical stratification, all prefectures in Japan were divided into 10 regions. The regions were then separated into 4 groups according to city size. Based on the population size of children aged 4 to 11 years in each region, 225 areas (205 city and 20 town or village) were established. Finally, 2,400 participants (8 to 16 participants/area) were allocated, representing the population composition by city size. The number of survey points and the number of samples by district obtained by the above sampling process are presented in the Supplemental Table. With reference to the national basic resident registry, participants were sampled from the specified survey areas by the equal interval sampling method. Participant extraction corresponded to the assigned age based on population composition in the respective areas. After mailing the survey request postcards, the questionnaires written in Japanese were hand-delivered and later collected by survey conductors using a drop-off and pick-up method, which has been validated to provide a higher response rate in population-based surveys [21]. In the survey of youths aged 4 to 11 years old, investigators also used the individual interview method in the presence of parents. Individuals who were capable of comprehending the Japanese language and completing the questionnaire in Japanese face-to-face with the investigator (including those with a family interpreter) were eligible. Of the selected 2,400 participants, 1,538 (64.1%) responded, of which 1,197 were elementary school students. Ethics approval for the use of analyzed data was obtained from the Sasagawa Sports Foundation. This study was approved by the institutional review board of Shinshu University School of Medicine (authorization number: 5416).

### PA among students

We evaluated the children’s preference for PA using an original questionnaire item simply asking, “Do you like physical activity?” The participants were asked to respond on a 4-point Likert scale of yes, somewhat yes, somewhat no, or no. In our analysis, we classified the answers as “like” (yes or somewhat yes) or “dislike” (somewhat no or no). Participation in school and club sports including lessons and league or team sports was identified using the following question for the parents of participants: “Is the child currently enrolled in school sports clubs, sports clubs as extracurricular activities, private sports clubs, or community sports clubs?” The parents answered “yes” or “no”. We assessed MVPA using the Japanese version of the WHO Health Behaviour in School-aged Children survey, one of the most comprehensive sources of data for school-aged student PA levels [22]. The validity of the Japanese version has already been verified in a study using accelerometers [23]. Before answering questions, a definition of PA was provided as “Physical activity is any activity that increases your heart rate and makes you out of breath some of the time. Physical activity can be done in sports, school activities, playing with friends, or walking or cycling to school. Some examples of physical activity are running, brisk walking, rollerblading, cycling, dancing, swimming, soccer, and basketball”. The participants were asked the following question: “Over the past 7 days, on how many days were you physically active for a total of at least 60 min per day?” The participants provided a number from 0 to 7 days (Supplemental figure).

### Covariates

The covariates in our models were selected a priori based on previously published literature [16–18, 24]. The participants were asked questions on gender, age, and grade. Their parents provided additional information on such sociodemographic factors as annual household income and family members by choosing the most suitable response from a set of predetermined options. Parents also answered questions regarding the exercise habits of themselves and their spouse (never, rarely, sometimes, and frequently) and their exercise habits with their children (never, rarely, sometimes, and frequently), as well as the children’s frequency of eating breakfast per week (every day, 5–6 days, 3–4 days, 1–2 days, or never), defecation frequency (every day, once every 2 days, once every 3 days, less than once every 3 days, or irregular), bedtime, wake-up time, and weekday screen time on electronic devices, including computers, tablets, smartphones, handheld gaming consoles, and television, during discretionary time (i.e., non-school use).

We classified the items of school grade as grade 1–2, grade 3–4, or grade 5–6, residential area as metropolitan area in Tokyo, ordinance-designated city area including 20 cities, city with a population of 100,000 or more, city with a population less than 100,000, or town/village, and annual household income as < 4,000,000 JPY, 4,000,000–7,999,999 JPY, or ≥ 8,000,000 JPY) [25], and family structure as two-generation household or household of three or more generations. We defined “skipping” as non-daily breakfast consumption or defecation. Based on the recommendations of the American Academy of Pediatrics, screen time was classified as < 2 h or ≥ 2 h [26]. Weekday sleep time was calculated from the reported bedtime and wake-up time and categorized as < 8 h or ≥ 8 h [17].

### Statistical analysis

We evaluated 2 main outcomes: participation in organized sports and MVPA level. MVPA level was categorized using the cut-off points of ≥ 5 days/week or ≤ 4 days/week based on MVPA reference values of at least 60 min/day in accordance with previous studies [1, 18, 23]. Considering the differences in gender for lifestyle and PA participation, Fisher’s exact test or the chi-square test were performed to compare variables between sexes. Multiple logistic regression analysis was performed on the factors related to the students who participated in organized sports. We also adopted multiple logistic regression analysis to identify factors related to adequate MVPA as dependent variables. Crude and adjusted odds ratios (ORs) and their 95% confidence intervals (CIs) were calculated while controlling for covariates. Spearman’s rank correlation coefficient was used to check for multicollinearity of covariates. Hosmer–Lemeshow testing was employed to assess the goodness-of-fit of the models. All statistical analyses were performed using SPSS statistical software version 28 (SPSS Inc., Chicago, Illinois). A *P* value of < 0.05 was considered statistically significant.

## Results

**Fig. 1:**
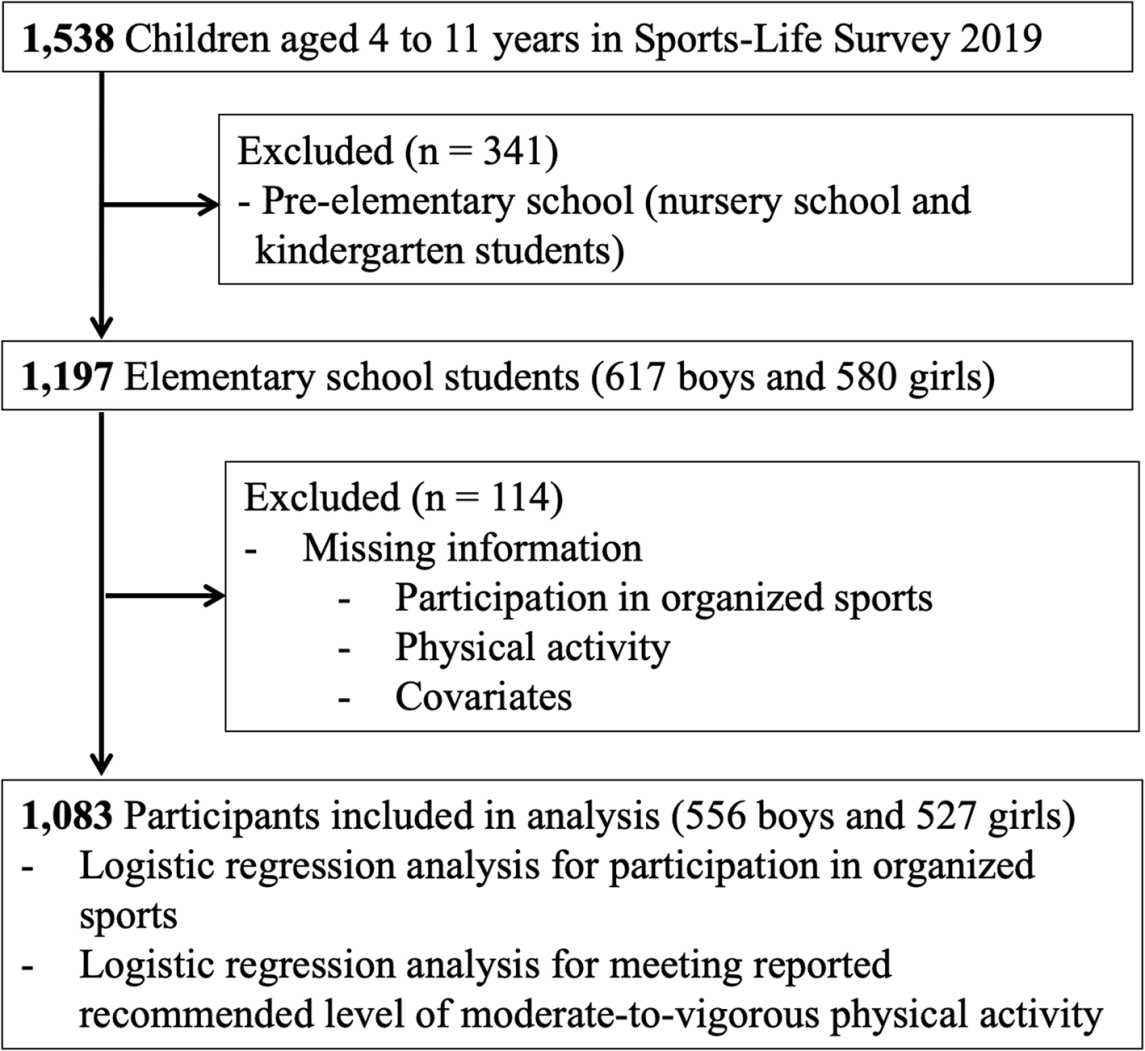
Study population selection flowchart

A total of 1,197 elementary school students and their parents were eligible for analysis (Fig. 1). Table 1 summarizes the participants’ characteristics, including socioeconomic background, lifestyle, and PA, according to gender. There was a similar proportion of boys (617 [51.5%]) and girls (580 [48.5%]). Significant differences were seen between the sexes for defecation and screen time habits, with constipation more common in girls (*P* = 0.008) and boys having longer weekday screen time (*P* = 0.034). Regarding PA, preference for PA, participation in organized sports, and more frequent MVPA were all significantly more common in boys (all *P* < 0.05). Although 1,053 (88.2%) students reported to like PA, 725 students (60.8%) actually participated in organized sports. Moreover, only 38 students (3.2%; 22 boys and 16 girls) responded with MVPA levels meeting WHO recommendations (≥ 60 min/day for 7 days/week). When interpreting recommended MVPA level as 60 min/day for ≥ 5 days/week as previously reported, 146 students (12.3%; 83 boys and 63 girls) met this criterion.

**Table 1:** Sociodemographic characteristics of participants by gender

Variable	Total	Boys	Girls	*P* value
	*n* = 1,197	*n* = 617	*n* = 580	
Grade, n (%)				0.62
Grade 1–2	328 (27.4)	164 (26.6)	164 (28.3)	
Grade 3–4	406 (33.9)	217 (35.2)	189 (32.6)	
Grade 5–6	463 (38.7)	236 (38.2)	227 (39.1)	
Residential area, n (%)				0.23
Metropolitan city	59 (4.9)	33 (5.3)	26 (4.5)	
Ordinance-designated city	217 (18.1)	99 (16.0)	118 (20.3)	
Population of 100,000 or more	495 (41.4)	251 (40.7)	244 (62.4)	
Population of less than 100,000	294 (24.6)	162 (26.3)	132 (22.8)	
Town/village	132 (11.0)	72 (11.7)	60 (10.3)	
Annual household income, n (%)				0.99
< 4,000,000 JPY	188 (20.3)	95 (20.1)	93 (20.4)	
4,000,000–7,999,999 JPY	572 (51.0)	291 (50.8)	281 (51.2)	
≥ 8,000,000 JPY	168 (15.0)	86 (15.0)	82 (14.9)	
Unknown	194 (17.3)	101 (17.6)	93 (16.9)	
Family structure, n (%)				0.33
Two-generation household	1,014 (85.0)	515 (84.0)	499 (86.0)	
Household of three or more generations	179 (15.0)	98 (16.0)	81 (14.0)	
Siblings, n (%)				0.62
No	176 (14.8)	87 (14.2)	89 (15.3)	
Yes	1017 (85.2)	526 (85.8)	491 (84.7)	
Father's participation in physical activity, n (%)				0.78
Never	234 (22.3)	118 (21.9)	116 (22.7)	
Rarely	368 (35.0)	189 (35.1)	179 (35.0)	
Sometimes	300 (28.5)	160 (29.7)	140 (27.3)	
Frequently	149 (14.2)	72 (13.4)	77 (15.0)	
Mother's participation in physical activity, n (%)				0.17
Never	373 (32.7)	180 (30.6)	193 (35.1)	
Rarely	474 (41.6)	258 (43.8)	216 (39.3)	
Sometimes	239 (21.0)	128 (21.7)	111 (20.2)	
Frequently	53 (4.7)	23 (3.9)	30 (5.5)	
Exercise habits with family, n (%)				0.13
Never	100 (8.4)	48 (7.8)	52 (9.0)	
Rarely	363 (30.5)	182 (29.7)	181 (31.3)	
Sometimes	586 (49.2)	297 (48.5)	289 (50.0)	
Frequently	142 (11.9)	86 (14.0)	56 (9.7)	
Breakfast consumption, n (%)				0.62
Skipping	69 (5.8)	38 (6.2)	31 (5.4)	
Daily	1,124 (94.2)	576 (93.8)	548 (94.6)	
Defecation, n (%)				0.008
Skipping	341 (28.8)	155 (25.4)	186 (32.4)	
Daily	843 (71.2)	455 (74.6)	388 (67.6)	
Weekday screen time, n (%)				0.034
< 2 h	780 (66.9)	386 (64.0)	394 (70.0)	
≥ 2 h	386 (33.1)	217 (36.0)	169 (30.0)	
Weekday sleep time, n (%)				0.78
< 8 h	55 (4.6)	27 (4.4)	28 (4.9)	
≥ 8 h	1,134 (95.4)	585 (95.6)	549 (95.1)	
Preference for physical activity, n (%)				0.009
Dislike	141 (11.8)	58 (9.4)	83 (14.3)	
Like	1053 (88.2)	557 (90.6)	496 (85.7)	
Organized sports participation, n (%)				< 0.001
No	468 (39.2)	197 (32.1)	271 (46.7)	
Yes	725 (60.8)	416 (67.9)	309 (53.3)	
MVPA level, ≥ 60 min/day				0.014
Never	531 (44.7)	249 (40.6)	282 (49.0)	
1 to 4 days/week	512 (43.1)	281 (45.8)	231 (40.1)	
5 to 7 days/week	146 (12.3)	83 (13.5)	63 (10.9)	

Data were missing on annual household income (*n* = 75), family structure (*n* = 4), father's physical activity (*n* = 146), mother's physical activity (*n* = 58), exercise habits with family (*n* = 6), breakfast consumption (*n* = 4), defecation habits (*n* = 13), weekday screen time (*n* = 31), weekday sleep time (*n* = 8), preference for physical activity (*n* = 3), organized sports participation (*n* = 4), and MVPA level (*n* = 8). MVPA, moderate-vigorous physical activity. The average (median) annual Japanese household income in 2018 was 5,523,000 JPY (4,370,000 JPY). The exchange rates on June 16, 2023, were 1 USD = 141 JPY and 1 EUR = 155 JPY

Table 2 shows the results of the logistic regression analysis conducted to determine the factors related to participating in organized sports. Due to a relatively strong correlation between the parents' own exercise habits and the frequency of exercising with their children (*r* > 0.3), the parents' own exercise habits were excluded from the covariates due to multicollinearity. Multivariate analysis revealed that male (OR 1.99, 95% CI 1.53–2.59 based on female, *P* < 0.001), higher grade (grade 5–6: OR 1.73, 95% CI 1.24–2.42 based on grade 1–2,* P* for trend = 0.001), residential area (population of less than 100,000: OR 0.43, 95% CI 0.21–0.88 and town/village: OR 0.37, 95% CI 0.17–0.80 based on metropolitan area, *P* for trend = 0.002), higher annual household income (4,000,000–7,999,999 JPY: OR 1.98, 95% CI 1.38–2.84 and ≥ 8,000,000 JPY: OR 3.22, 95% CI 1.99–5.20, based on < 4,000,000 JPY, *P* for trend = 0.003), and more opportunities to exercise with their parents (sometimes: OR 2.36, 95% CI 1.46–3.83 and frequently: OR 3.33, 95% CI 1.81–6.11 based on “never”, *P* for trend < 0.001) were significantly associated with participation in organized sports. Having breakfast daily (OR 2.77, 95% CI 1.52–5.04 based on skipping, *P* < 0.001) and shorter weekday screen time (< 2 h: OR 1.45, 95% CI 1.09–1.93 based on ≥ 2 h, *P* = 0.002) were also significantly related to participation in organized sports.

**Table 2:** Correlations for participation in organized sports and MVPA in Japanese children

Variable	Total (*n* = 1,083)	Participation in organized sports				MVPA level ≥ 60 min/day for ≥ 5 days/week			
		n (%)	Univariate	Multivariate	*P* value	n (%)	Univariate	Multivariate	*P* value
			OR (95% CI)	OR (95% CI)			OR (95% CI)	OR (95% CI)	
Gender									
Female (reference)	527	280 (53.1)	1.00	1.00		60 (11.4)	1.00	1.00	
Male	556	377 (67.8)	**1.86 (1.45–2.38)**	**1.99 (1.53–2.59)**	** < 0.001**	78 (14.0)	1.27 (0.89–1.82)	1.20 (0.83–1.74)	0.30
Grade									
Grade 1–2 (reference)	302	165 (54.6)	1.00	1.00		33 (10.9)	1.00	1.00	
Grade 3–4	371	236 (63.6)	**1.45 (1.07–1.98)**	**1.51 (1.09–2.10)**		43 (11.6)	1.07 (0.66–1.73)	1.04 (0.64–1.70)	
Grade 5–6	410	256 (62.4)	**1.38 (1.02–1.87)**	**1.73 (1.24–2.42)**		62 (15.1)	1.45 (0.93–2.28)	1.56 (0.97–2.50)	
*P* value for trend	NA	NA	NA	**0.001**		NA	NA	**0.043**	
Residential area									
Metropolitan area (reference)	55	42 (76.4)	1.00	1.00		7 (12.7)	1.00	1.00	
Ordinance-designated city area	197	122 (61.9)	**0.50 (0.25–0.99)**	0.59 (0.28–1.22)		16 (8.1)	0.61 (0.24–1.56)	0.61 (0.23–1.64)	
Population of 100,000 or more	441	273 (61.9)	**0.50 (0.26–0.97)**	0.53 (0.27–1.06)		57 (12.9)	1.02 (0.44–2.36)	1.02 (0.43–2.41)	
Population of less than 100,000	268	156 (58.2)	**0.43 (0.22–0.84)**	**0.43 (0.21–0.88)**		43 (16.0)	1.31 (0.56–3.09)	1.27 (0.53–3.07)	
Town/village	122	64 (52.5)	**0.34 (0.17–0.70)**	**0.37 (0.17–0.80)**		15 (12.3)	0.96 (0.37–2.51)	0.96 (0.36–2.57)	
*P* value for trend	NA	NA	NA	**0.002**		NA	NA	0.165	
Annual household income									
< 4,000,000 JPY (reference)	179	77 (43.0)	1.00	1.00		22 (12.3)	1.00	1.00	
4,000,000–7,999,999 JPY	558	351 (62.9)	**2.25 (1.60–3.16)**	**1.98 (1.38–2.84)**		73 (13.1)	1.07 (0.65–1.79)	0.88 (0.52–1.50)	
≥ 8,000,000 JPY	166	122 (73.5)	**3.67 (2.33–5.79)**	**3.22 (1.99–5.20)**		23 (13.9)	1.15 (0.61–2.15)	0.96 (0.50–1.84)	
Unknown	180	107 (59.4)	**1.94 (1.28–2.95)**	**1.87 (1.20–2.91)**		20 (11.1)	0.89 (0.47–1.70)	0.77 (0.40–1.49)	
*P* value for trend	NA	NA	NA	**0.003**		NA	NA	0.58	
Exercise habits with parents									
Never (reference)	92	36 (39.1)	1.00	1.00		8 (8.7)	1.00	1.00	
Rarely	325	180 (55.4)	**1.93 (1.20–3.10)**	1.55 (0.94–2.54)		30 (9.2)	1.07 (0.47–2.42)	1.11 (0.48–2.54)	
Sometimes	538	347 (64.5)	**2.93 (1.79–4.45)**	**2.36 (1.46–3.83)**		71 (13.2)	1.60 (0.74–3.44)	1.65 (0.75–3.64)	
Frequently	127	94 (73.4)	**4.30 (2.42–7.63)**	**3.33 (1.81–6.11)**		29 (22.7)	**3.08 (1.34–7.09)**	**2.89 (1.22–6.88)**	
*P* value for trend	NA	NA	**NA**	** < 0.001**		NA	NA	**0.001**	
Breakfast consumption									
Skipping (reference)	60	20 (33.3)	1.00	1.00		7 (11.7)	1.00	1.00	
Daily	1023	637 (62.3)	**3.30 (1.90–5.73)**	**2.77 (1.52–5.04)**	** < 0.001**	131 (12.8)	1.11 (0.50–2.50)	0.93 (0.39–2.18)	0.798
Defecation frequency									
Skipping (reference)	317	182 (57.4)	1.00	1.00		29 (9.1)	1.00	1.00	
Daily	766	475 (62.0)	1.21 (0.93–1.58)	0.92 (0.69–1.23)	0.596	109 (14.2)	**1.65 (1.07–2.54)**	1.43 (0.92–2.24)	0.132
Weekday screen time									
≥ 2 h (reference)	355	188 (53.0)	1.00	1.00		32 (9.0)	1.00	1.00	
< 2 h	728	469 (64.4)	**1.61 (1.24–2.08)**	**1.45 (1.09–1.93)**	**0.002**	106 (14.6)	**1.72 (1.13–2.61)**	**1.67 (1.08–2.59)**	**0.021**
Weekday sleep time									
< 8 h (reference)	51	28 (54.9)	1.00	1.00		9 (17.6)	1.00	1.00	
≥ 8 h	1032	629 (60.9)	1.28 (0.73–2.26)	1.10 (0.58–2.09)	0.882	129 (12.5)	0.67 (0.32–1.40)	0.69 (0.31–1.52)	0.269

*MVPA* Moderate-to-vigorous physical activity, *OR* Odds ratio, *CI* Confidence interval, *NA* Not applicable

Meeting an MVPA level of 60 min/day for ≥ 5 days/week was significantly associated with having frequent exercise habits with parents (frequently: OR 2.89, 95% CI 1.22–6.88 based on “never”, *P* for trend = 0.001) and shorter weekday screen time (< 2 h: OR 1.67, 95% CI 1.08–2.59 based on ≥ 2 h, *P* = 0.021).

## Discussion

In this study, 60.8% of the elementary school respondents participated in school or club sports, such as lessons and league or team sports. Being male, in the upper grades, living in a city, having a higher household income, having more opportunities to exercise with parents, and having lower screen time were all significantly associated with participation in organized sports. We witnessed that only 12.3% of students engaged in regular MVPA levels, which was related to the habit of exercising with their parents and lower screen time. These findings indicated a need for greater parental involvement to increase PA levels in elementary school-aged children.

The reduced PA and increased sedentary time in recent decades have emphasized the need to promote opportunities for sport participation in elementary school-aged children in order to increase activity levels [1–3, 17]. In children and adolescents, PA provides benefits towards physical fitness, cardiometabolic health, bone health, cognitive outcome, mental health, and reduced adiposity [1–3]. In addition, participating in organized sports helps form psychosocial growth and social identity. Sports involvement is also associated with improved teamwork, social skills, self-esteem, and such educational and occupational skills as determination, perseverance, grit, resilience, and critical thinking [27]. The majority of parents believe that sports will benefit their children academically and improve their future careers as well [28]. Moreover, 73% of adults who played sports were found to have also done that sport when they were younger [28], supporting that PA habits are formed during childhood and can remain for life.

Social, family, and lifestyle factors have all been reported as determinants of exercise habits among schoolchildren, which agreed with the results of this study [17, 18, 21, 25]. Participation in organized sports has been associated with higher annual income and residence in urban areas [29]. In order for children to join organized sports, a significant financial outlay may be needed for uniforms, equipment, tournament entry fees, travel expenses, and more [2]. The results of the present study also showed household income to be associated with children's participation in organized sports. Thus, prohibitively high cost may be a barrier to participation for some sports. Children living in densely populated areas may be more likely to participate in organized sports because of more sports facilities and grounds around them and easier access [30].

In spite of participation in organized sports, many children still did not reach MVPA levels that meet WHO guidelines. We observed that while 60.8% of children participated in organized sports, only 12.3% reported MVPA of 60 min or more ≥ 5 days/week despite most expressing a like for PA (88.2%). This finding indicated incongruencies among liking sports, participating in organized sports, and actually doing physical exercise. Nonetheless, children who were engaged in organized sports were more likely to achieve PA guidelines and spent more time in MVPA than those who did not participate in organized sports [29]. Our study showed that less screen time and more exercise habits with parents were associated with both organized sports participation and MVPA frequency in children, and might be important factors driving those outcomes.

Students with longer television viewing time spent less time in club sports, indicating less engagement in overall PA [31, 32]. Those results were consistent with our own that individuals with shorter screen time spent significantly more time participating in PA. The association between sedentary behavior and adverse health outcomes is generally stronger for television viewing or recreational screen time as the specific exposure variable than for total sedentary time in youths [33]. Previous studies have indicated that high screen time during childhood is an accurate predictor of objectively assessed low PA [34]; however, the potential of screen time reduction as a component in interventions aiming to increase MVPA seems limited [33]. Nevertheless, several reports have indicated an indirect relationship between TV viewing and metabolic and cardiovascular risk in young people [35–37]. Despite a weak relationship with MVPA, efforts made to limit screen time may therefore have important public health implications.

Frequent exercise with parents most strongly influenced whether their children participated in organized sports or engaged in frequent MVPA in this study. Parental support includes various means, including participation in PA jointly with children, providing logistic support (e.g., transportation), acting as role models, and encouraging children to do PA [38–41]. Hong et al. demonstrated that children and adolescents meeting the WHO MVPA guidelines were positively associated with several kinds of parental support, such as involvement, encouragement, modeling, and financial resources, and that parental MVPA was linked to that of their children aged 1–6 years [42]. Elsewhere, parental role modeling also had a strong effect on children’s PA [43], although it was noteworthy that inactive parents could overcome their lack of role modeling through effective encouragement [44]. Sawa et al. reported that Japanese elementary school students who lacked parental communication had a significant preponderance to both dislike and lack PA [17]. The authors also emphasized the importance of parental involvement in intervention strategies to increase the child’s PA.

This study had several limitations. First, recall bias might have been present since the responses from children were used to determine PA level. We therefore used an accelerometer-validated PA questionnaire to increase the accuracy of PA level assessment [23]. In contrast, the information on organized sports participation was based on parent responses and thus considered more reliable. Second, there might have been selection bias due to missing data through unreturned questionnaires. Third, the survey used in this cross-sectional study was conducted from June to July, which corresponds to the beginning of summer in Japan, although it is necessary to consider the effects of seasonality, weather, and geographical differences during that period. Longitudinal approaches throughout the year should be incorporated to more clearly assess PA. Fourth, the participants were randomly selected without consideration of possible underlying medical conditions. The survey may have included children with medical conditions that restricted PA; however, this cohort can be considered as a representative population, and not a special group. Finally, we could not account for the effects of unmeasured factors, including anthropometric data and such health information as comorbidities and dietary habits, which also might have influenced involvement in PA.

The present survey was conducted from June to July 2019 and might differ from the current post-Coronavirus disease 2019 (COVID-19) pandemic situation. However, as children's PA levels are expected to gradually return to pre-COVID-19 levels [45], the findings of this study are considered useful as they are reflective of the typical situation of normal children.

## Conclusion

Our study revealed several social, family, and lifestyle determinants of participation in organized sports and meeting published MVPA levels in Japanese elementary school-aged schoolchildren. Parental involvement appears to have a strong influence on children’s engagement in PA and should be encouraged to increase activity levels.

## Supplementary Information

**Additional file 1:** **Supplemental Table.** Number of survey points and samples by region/city size.

**Additional file 2:** **Supplemental figure.** Questionnaire on physical activity.

## Data Availability

The data that support the findings of this study are available from the Sasagawa Sports Foundation but restrictions apply to the availability of these data, which were used under permission for the current study, and so are not publicly available. Data are however available from the corresponding author (Noriko Motoki: nmotoki@shinshu-u.ac.jp) upon reasonable request and with permission of the Sasagawa Sports Foundation.

## Abbreviations

CI: Confidence interval
COVID-19: Coronavirus disease 2019
MVPA: Moderate-to-vigorous physical activity
OR: Odds ratio
PA: Physical activity
WHO: World Health Organization
