# Supplementary material for: Familial and lifestyle factors related to physical activity in elementary school students: a cross-sectional study based on a nationally representative survey in Japan
Source: BMC Pediatr. 2023 Jul 4;23:338. doi: 10.1186/s12887-023-04162-3 (PMC10318804; doi:10.1186/s12887-023-04162-3)
Supplement: Supplementary file 1 — Additional file 1: Supplemental Table. Number of survey points and samples by region/city size. [file 12887_2023_4162_MOESM1_ESM.docx]

| Supplemental Table. Number of survey points and samples by region/city size | | | | | | |
| --- | --- | --- | --- | --- | --- | --- |
|  | | City size | | | | Total |
|  |  | Metropolitan city | Population of 100,000 or more | Population of less than 100,000 | Town/village |  |
| Region | Hokkaido | 3 (34) | 2 (25) | 2 (16) | 2 (16) | 9 (91) |
|  | Tohoku | 2 (20) | 6 (62) | 5 (51) | 2 (25) | 15 (158) |
|  | Kanto | 29 (306) | 32 (340) | 10 (107) | 3 (33) | 74 (786) |
|  | Hokuriku | 1 (15) | 4 (40) | 4 (37) | 1 (7) | 10 (99) |
|  | Tosan | – | 3 (37) | 4 (42) | 2 (16) | 9 (95) |
|  | Tokai | 6 (71) | 11 (116) | 5 (54) | 2 (18) | 24 (259) |
|  | Kinki | 11 (114) | 17 (179) | 8 (80) | 2 (21) | 38 (394 ) |
|  | Chugoku | 3 (40) | 6 (69) | 2 (26) | 1 (10) | 12 (145) |
|  | Shikoku | – | 4 (40) | 2 (21) | 1 (10) | 7 (71) |
|  | Northern Kyushu | 6 (65) | 9 (103) | 8 (90) | 4 (44) | 27 (302) |
| Total | | 61 (655) | 94 (1,011) | 50 (524) | 20 (200) | 225 (2,400) |
|  | | |  |  |  |  |

Data are expressed as number of survey points (sample number).
