# Supplementary material for: Familial and lifestyle factors related to physical activity in elementary school students: a cross-sectional study based on a nationally representative survey in Japan
Source: BMC Pediatr. 2023 Jul 4;23:338. doi: 10.1186/s12887-023-04162-3 (PMC10318804; doi:10.1186/s12887-023-04162-3)
Supplement: Supplementary file 2 — Additional file 2: Supplemental figure. Questionnaire on physical activity. [file 12887_2023_4162_MOESM2_ESM.docx]

**Supplemental figure: Questionnaire on physical activity**

**English version**

Physical activity is any activity that increases your heart rate and makes you out of breath some of the time. Physical activity can be done in sports, school activities, playing with friends, or walking or cycling to school. Some examples of physical activity are running, brisk walking, rollerblading, cycling, dancing, swimming, soccer, and basketball.

Over the past 7 days, on how many days were you physically active for a total at least 60 minutes per day?

Please select the number of days from 0 to 7.
